# Production and Optimization of Biodiesel in a Membrane Reactor, Using a Solid Base Catalyst

**DOI:** 10.3390/membranes12070674

**Published:** 2022-06-30

**Authors:** Olusegun Ayodeji Olagunju, Paul Musonge, Sammy Lewis Kiambi

**Affiliations:** 1 Chemical Engineering Department, Durban University of Technology, Durban 4000, South Africa; 2 Institute of Systems Science, Durban University of Technology, Durban 4000, South Africa; paulm@dut.ac.za; 3 Faculty of Engineering, Mangosuthu University of Technology, Durban 4000, South Africa; 4 Chemical and Metallurgical Department, Vaal University of Technology, Private Bag X021, Vanderbijlpark 1911, South Africa; sammyk1@vut.ac.za

**Keywords:** soybean oil methyl ester, alternative fuels, membrane reactor, response surface methodology, central composite design, fuel characteristics

## Abstract

The commercial Calcium oxide was successfully embedded on activated carbon surfaces to increase the reactive surface area of a composite catalyst material CaO/AC. The composite catalyst material was also successfully packed in the tubular titanium dioxide/Aluminum dioxide ceramic membrane reactor used to separate the biodiesel produced. Virgin soybean oil was used as precursor feedstock for the reaction. Using a central composite approach, response surface methodology (RSM) was employed to obtain the optimum conditions for producing biodiesel from soybean oil. A total of four process factors were examined (2^4^ experimental designs). 30 experiments were derived and run to investigate the effects of temperature, reaction time, methanol to oil molar ratio, and catalyst concentration (calcium oxide attached on activated carbon). 96.9 percent of soybean oil methyl ester (SOME/biodiesel) was produced at 65 °C temperature, 90 min of reaction time, 4.2:1 molar ratio of methanol to oil, and 3.0 wt.% catalyst concentration. The measured yield and expected biodiesel production values were correlated in a linear sequence. The fuel qualities of SOME/biodiesel were tested, including kinematic viscosity, density, flash point, copper corrosion, calorific value, cloud point, pour point, ash content, and carbon residue.

## 1. Introduction

Non-renewable energy sources such as oil and gas are going depleted all across the globe very rapidly owing to the growing in demand. Several nations in the globe are in quest of alternate sources of fuel for their energy demand. Due to its non-toxic nature, biodiesel is a viable option to petroleum diesel because of its renewability as well as its reduced pollution of Carbon monoxide, Sulphur dioxide, and particulate matters [1]. Vegetable oils, greases, and even animal fats are feedstock for producing biodiesel. Biodiesel consist of fatty acid methyl ester (FAME), made from different plant based monomers. Different mechanisms of FAME synthesis have been developed by researchers in an attempt to determine a sustainable and efficient way of producing biodiesel as an alternative source of energy material.

**Figure 1:**
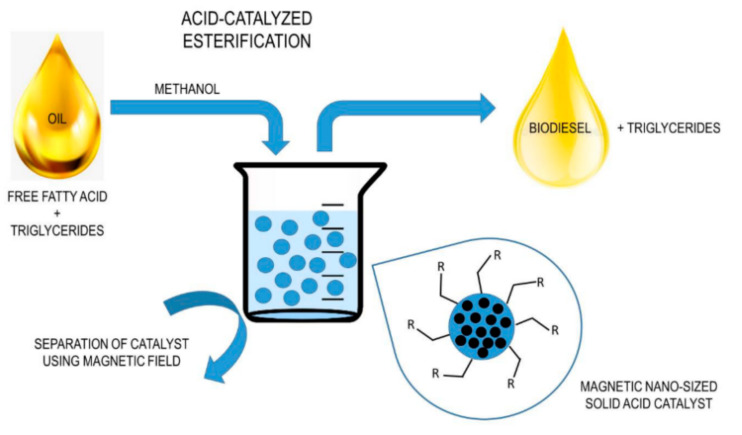
Biodiesel synthesis process using a magnetized solid acid-catalyzed esterification process [2].

Figure 1 shows preliminary work carried out to produce biodiesel from triglycerides through acid-catalyzed esterification. The eminent application is an advanced technology that makes use of magnetic nanosized solid acid catalyst for both esterification and magnetic separation to produce biodiesel. This method complements the membrane reactor biodiesel production using solid base catalyst in this work. Our method and approach is characteristically a trans-esterification mechanism that bypasses the esterification process because of the low free fatty acid content of the feedstock as a result of it being a virgin oil.

With the increased concentration of an acid or base accelerator in the reaction, triglycerides can be transesterified with alcohol (usually methanol) to produce fatty acid methyl ester (FAME) [3,4]. Soyabean oil is one of the approved source materials for producing biodiesel in South Africa and this serves as the basis for the use of the feedstock in this study. The government had approved large fertile land sites for the plantation of soya bean feedstock in Eastern Cape Province specifically for biodiesel production, thus eliminating the food-fuel debates [5].

The traditional technique has been used to produce biodiesel for the majority of its history. This entails the reaction of the feedstock with methanol and having some homogenous base catalysts mainly NaOH or KOH present during the process [4,6]. Base promoters such as sodium hydroxide or potassium hydroxide have several drawbacks, including lather generation and difficulties in recovery, which later results in the use of water for purification and, as a result, increased costs for producing biodiesel. The overall cost of making biodiesel with the use of homogeneous catalyst is not economically, nor profitable as compared to the total cost of making gasoline from fossils [7]. Consequently, it is necessary to examine an alternate method that has a lower level of corrosive nature, which results in cleaner, more efficient, and environmentally benign operations, as well as the simplicity with which the promoter (catalyst) can be removed from the final product.

**Figure 2:**
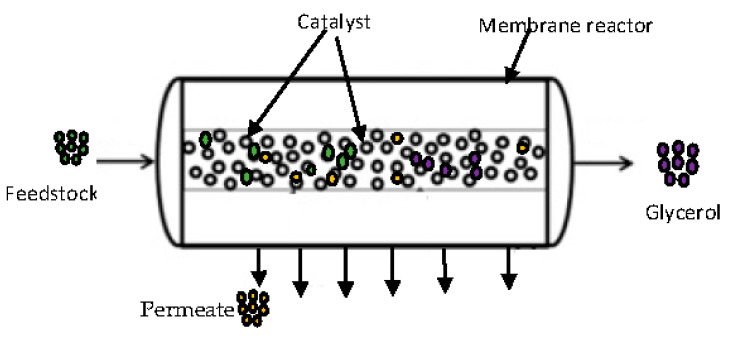
A schematic diagram showing the feedstock and membrane-catalyst interactive surface.

Heterogeneous catalysis with the use of a membrane reactor is an alternative process that can be used to overcome the challenges encountered by the homogenous process. In this process, the promoter is effortlessly recovered and then recycled. Figure 2 shows the feedstock introduced into the membrane reactor where reaction and separation take place as an integrated process, which does not require further washing and recycle constraints, leading to an increased conversion of feedstock to biodiesel due to enhanced interaction between the reactants within the membrane–catalyst interphase [8].

Various studies have explored substantially the use of membrane advanced technologies in filtration process and for treating wastewater due to its capabilities of separating different elements in a singular process unit on the basis of molecule size. This method involves the reaction and separation of two or more components in a singular process stream, thereby eliminating the use of water in the entire process. Membrane reactor has the capability of selectivity by ensuring that only components with less molecular sizes passes through and then holding back the components with high molecular sizes. In addition, this technique enhances interaction between the insoluble feedstock (oil) and the solid promoter (catalyst), thereby yielding maximum product [9].

As South Africa is a water-deficient nation, adapting the membrane technologies in the production of biodiesel will save water by eliminating the need for purification and then wastewater treatment in the process. This also has cumulative advantage on the environment has it will be free from pollution. A prior study was conducted by [10] used KOH as reaction promoter and palm oil as feed source but the issue of by-selectivity and product (gylcerine) still finding its way into the product stream was not addressed. Therefore, this study’s goal is to address the issue of membrane permeability(selectivity) and further downstream purification. 

To find a solution to this problem, an estimation of the dispersed oil droplets size found in the permeate stream was carried out and then a suitable membrane pore size was selected. The minimum particle size in the oil-methanol emulsion can be estimated from the work of [11] which showed that the average drop size for unreacted oil was 44 microns with a lower and upper size limit of 12 and 400 microns, respectively [11]. Based on this finding, a membrane of 0.02 microns was selected for the current work, which was able to trap the unreacted oil within the membrane and allowed only biodiesel and methanol to pass through it. The retention of free glycerol and unreacted oil in the reaction medium micro-filtrated by the 0.02 μm membrane eliminates the use of water in the process, water is conserved for other purposes and therefore reduced the production cost. 

Furthermore, research into the optimization process is critical for the development of producing biodiesel. Biodiesel process was previously performed cautiously by varying one element at a time, and the output is a dependency of a singular parameter, which is time consuming and costly [12]. This approach does not incorporate interaction influences between the independent parameters and does not reflect the whole influence of the parameters on the processes [13]. Meanwhile, the utilization of the response surface methodology (RSM) approach in a multidimensional system gives a stepwise approach in examining the interactive tendencies of the factors by implementing statistical technique.

The experimental design of biodiesel production which is designed utilizing RSM can predict the reactions beneath diverse transesterification scenarios with accurate error estimates. This is important when high volume of biodiesel production is required. 

Studies conducted by [14], showed that RSM was employed in the optimization process of the methyl ester production using sunflower as the feedstock. Similarly, [15] used the same approach to produce biodiesel from J. curcas oil, which contained a high concentration of free fatty acids (FFA). Furthermore, RSM was also used to improve the base-catalyzed conditions for biodiesel synthesis while using oil from marula seed as source oils [16,17]. To enhance quality of biodiesel from soybean oil in a membrane reactor, research efforts have been made in the current study to improve the process parameters for transesterification reaction. The effect of many factors on transesterification, such as temperature, reaction duration, molar ratio, and catalyst concentration, has been investigated in detail. The results of the qualitative tests performed on the soybean oil methyl esters were also presented.

In this work, we mitigated the problem of insolent seepage of glycerol and unreacted oil molecules across the membrane, into the permeate collector reservoir thereby contaminating the biodiesel and methanol separated as target products of the reaction. This trans-membrane contamination demands an additional step of washing off the contaminants using water in a rigorous and expensive process. The preeminent innovative feature of this work is the engineering assembly and design of the membrane reactor that has a characteristic feature that makes use of a micro-filtrated membrane with a super reduced pore size of 0.02 μm modified with nanocatalyst materials fortifying and enhancing the surface reaction interphases. The resultant effect is a synergistic mechanism of efficient retention of the free glycerol and the unreacted oil in the reaction medium with savage decomposition and molecular restructuring of unreacted materials into biodiesel and methanol at the filtration membrane junction. The membrane matrix nanocatalyst impregnation serve both increasing the reactive surfaces and assist even narrowing the pores on the filtration membrane.

## 2. Experimental

### 2.1. Materials

**Figure 3:**
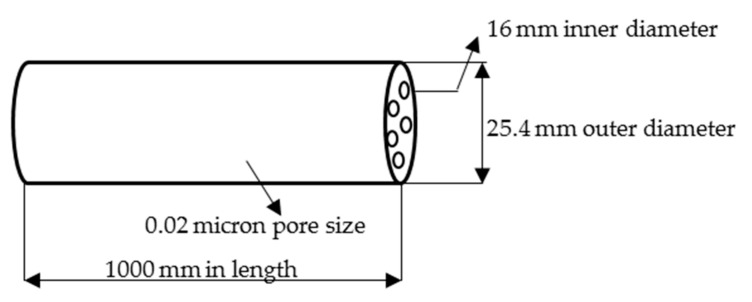
TiO_2_/Al_2_O_3_ tubular ceramic membrane and its specifications.

Feedstock (Soyabean oil) was obtained from a neighborhood store. Laboratory supplies company, Durban, South Africa, supplied the lab use methanol (99.8 percent). Commercialize calcium oxide (98.9 percent) and activated carbon employed as promoter and support respectively, were supplied by Associated Chemical Enterprise, Durban, South Africa. A tubular TiO_2_/Al_2_O_3_ tubular ceramic membrane purchased from Atech Innovations Gmbh, Wiesenbusch, Germany served as the reaction and separation media. Figure 3 shows the membrane’s dimensions of 1000 mm, 16 mm, 25.4 mm, and 0.02 m in length, inner and outer diameter, and pore size respectively. 

### 2.2. Synthesis and Evaluation of Catalysts

The catalysts mixture was made by dispersing the Calcium oxide in demineralized water and mixing it thoroughly. In order to remove particles and debris from the activated carbon, it was rinsed with demineralized water before being oven-dried at 100 degrees Celsius for 24 h, thereafter, left in a desiccator to cool-off and kept in a container. Activated carbon was introduced to the Calcium oxide solution, and then stirred in a shaker at 150 rpm for a day at a temperature of 25 degrees Celsius. It was observed that the total quantity of adsorbed CaO unto the surface of the activated carbon was 40.50 percent by weight based on prior weight of activated charcoal, which was calculated gravimetrically [18]. 

**Figure 4:**
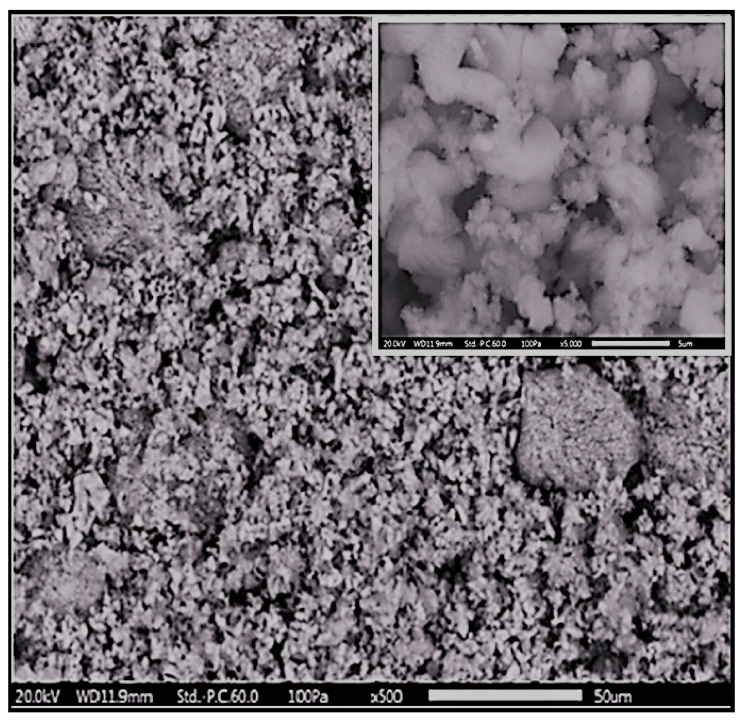
Calcium oxide on activated carbon (CaO/AC) as seen under a scanning electron Microscope. The insert shows the CaO particles heterogeneously dispersed over the activated carbon.

In addition, the attributes of the produced supported promoter were ascertained. Images captured with an FEI Quanta 200 FESEM (Oregon, USA) scanning electron microscope were used to create scanning electron micrographs (SEM) as shown in Figure 4. The peak amplitude was 20 kV at the time of the experiment. ETD and Low kV SSBSED (Oregon, USA) detectors were used for the SE and BSE detection. Adsorption of nitrogen at 77 degrees Celsius was conducted using ASAP 2020, Micromeritics (Atlanta, USA) in order to determine the specific surface area and pore volumes. For the purpose of collecting adsorption data, degassing at 120 degrees Celsius with a residual pressure of 300 μm Hg for 24 h was carried out using the degassing port [18].

### 2.3. Transesterification Process in Membrane Reactor

**Figure 5:**
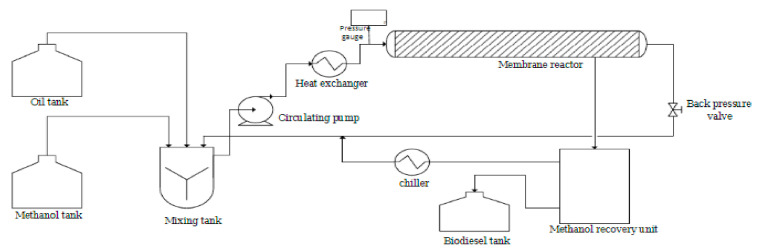
A membrane reactor for biodiesel synthesis is depicted schematically in this diagram [18].

Methanol and the feedstock (soyabean oil) were added to the mixing tank one at a time. Oil to methanol volume ratios ranged from 3:1 to 6:1, and CaO/AC was loaded into the membrane reactor. As shown in Figure 5, methanol was continually injected into the reactor through a circulating pump, as well as the heat exchanger was turned on to preheat the reaction medium. 

Following that, the reactants were fed into the reaction medium. Two pressure gauges were used to measure the pressure inside the membrane, which was maintained at a constant 100 Kpa. The beaker was used to receive the permeates comprising of biodiesel and methanol. The circulating pump and heat exchanger were turned off at the end of every cycle. Following that, the catalysts were removed, and the reactor was purged with methanol for half an hour before being completely emptied. The following equation (1) was used to compute the biodiesel production in this study:

Biodiesel produced (%) = (weight of biodiesel produced/weight of feedstock required) × 100%(1)

### 2.4. Design of Experiments

Transesterification variables were analyzed using response surface methodology (RSM) employing the central composite design (CCD) with four components altered at three levels, each of which had an influence on the produced biodiesel. A high level, denoted as (+1), a low level denoted as (−1) and a middle point (0) and there were 30 trials in all. The design variables were temperature (X_1_, °C), reaction time (X_2_, min), the molar ratio (X_3_), and catalyst concentration (X_4_) whereas the response variable was the amount of biodiesel produced (Y, percent). Table 1 shows the range and values of the independent variables that were selected for the current study, for each investigation, three replicates were carried out, and the average biodiesel yield signifies the response variable, denoted as Y.

### 2.5. Analytical Statistics (ANOVA)

A multiple regression methodology was then used to apply the polynomial equation scaled to the power of two to the collected data. As a result, adhering to an empirical model that explains the relationship between outcomes assessed against the experiment’s independent variables. A four-factor approach was used to develop the empirical predictive model, represented as:

Y = α0 + α1X_1_ + α2X_2_ + α3X_3_ + α4X_4_ + α12X_1_X_2_ + α13X_1_X_3_ + α14X_1_X_4_ + α23X_2_X_3_ + α24X_2_X_4_ + α34X_3_X_4_ + α11X_1_^2^ + α22X_2_^2^ + α33X_3_^2^ + α44X_4_^2^ (2)

where Y is the predicted response, α0 is the intercept, α1, α2, α3, α4 are linear coefficients, α11, α22, α33, α44 are squared coefficients, and α12, α13, α14, α23, α24, α34 are interaction coefficients and X_1_ denoted temperature (°C), X_2_ was reaction time (min), X_3_ was molar ratio and X_4_ was catalyst concentration. The response of the CCD design was fitted with a second-order polynomial equation. Statistical analysis of the data was performed by Design-Expert version 10.0 (Stat Ease, Inc., Minneapolis, MN, USA) to evaluate the analysis of variance (ANOVA), to determine the statistical significance of each term in the equation, the F value is more than 95% and the *p*-value is less than 0.05. By examining the response surfaces and solving the regression model equation, the optimal values for the selected variables were determined. To demonstrate the primary interacting impacts of the independent variables, the adjusted polynomial equation was expressed in the form of a three-dimensional response surface plots.

## 3. Results and Discussion

### 3.1. Characteristics of Catalysts

The investigation using scanning electron microscopy (SEM) was carried out. The images obtained from the SEM analysis revealed as shown in Figure 4 that the CaO/AC catalyst has a porous layered surface with active sites, irregularly shaped particles of varying sizes, and this suggests that the catalyst has a larger surface area on which reactions might take place.

In addition, the surface area, pore volume and pore width characterization of the supported CaO/AC catalyst were carried out and the result is shown in Table 2. The considerable decrease in BET surface area of the virgin activated carbon, which had 1425 m^2^/g, to the CaO/AC catalyst with 40.50 wt.% loading, which had 240.51 m^2^/g, indicating that calcium oxide particles have filled the pores of the catalyst support. The CO_2_ temperature programmed desorption (TPD) technique was utilized in order to ascertain the degree of basicity possessed by the catalyst. 

### 3.2. Experimental Design Based on Central Composite Design

The present work used a central composite design (CCD) to build an experimental matrices of independent reaction parameters such as temperature, reaction time, methanol to feedstock ratio, and catalyst concentration in order to maximize biodiesel yield. The produced biodiesel ranged from 48 percent to 96 percent. The polynomial equation containing the coefficient of the whole regression model equation was obtained using advanced multiple regression analysis, and its statistical significance was established. The significant parameters generated from the model in coded form have the following expression:

Y = 94.75 + 5.18X_1_ + 3.60X_2_ − 7.07X_3_ + 4.24X_4_ + 1.39X_1_X_2_ − 0.64X_1_X_3_ − 3.98X_1_X_4_ + 4.36X_2_X_3_ + 3.02X_2_X_4_ − 3.73X_3_X_4_ − 9.38X_1_^2^ − 1.50X_2_^2^ − 9.00X_3_^2^ − 3.63X_4_^2^ (3)

where Y is biodiesel yield and X_1_, X_2_, X_3,_ and X_4_ were the coded forms of temperature (°C), reaction time (min), methanol: oil ratio, catalyst concentration respectively. According to the equation, the coefficients with single factor represents the influence in a singular form, whereas the coefficients with two variables and 2nd order represents the interactions between itself and other related parameters. Using the negatively and positively symbols suffix (±), to distinguish between synergistic and antagonistic impacts, the positive character represents a synergistic impact, and the negative character represents antagonistic impact [19]. ANOVA (analysis of variance) was then used to determine the fitness of the model, with the least square methodology being used to calculate this fitness score. Table 3 and Table 4 reflect the results of the investigation of this design variant.

The ANOVA statistical analysis of the regression equation revealed that the R-squared value was 0.9574 (R-square value more than 0.75 shows that the model is fit for purpose). According to the calculated result, the overall variance of the data analyzed by the model can be explained by 95.74 percent of the total variation in the experimentally observed variables and associated interrelationships. The theoretical values of adjusted R-square and the predicted R-square were 0.9176 and 0.8861 respectively, the disparity between both the adjusted R-square and the predicted R-square is less than 0.2, indicating that the model is suitable.

The spectrum of R-squared is from 0 to 1, and a number that is close to 1 indicates that the model is more accurate. The term “adequate precision” (AP) refers to a proportion of the experimental signal-to-noise ratio [20]; an AP greater than 4 implies that the model will provide a satisfactory performance in predictions. The model’s appropriate precision value is 14.726, and the model’s C.V percent value of 6.49 confirms that the model is both flexible and reliable [21]. According to the model’s F value of 24.05, the model is statistically significant. The model’s *p*-value was less than 0.0001 (*p* less than 0.05), which indicates that it is statistically significant, while the lack of fit model was judged to be insignificant. The less significant the *p* value, the more significant the mutual interactions between the factors and, consequently, the more important those factors are in the model [22]. As a result, based on the *p*-value attained in the current investigation, it was discovered that the X_1_, X_2_, X_3_, X_4_, X_1_X_4_, X_2_X_3_, X_2_X_4_, X_3_X_4_, X_1_^2^, X_3_^2^, and X_4_^2^ were found to be significant.

**Figure 6:**
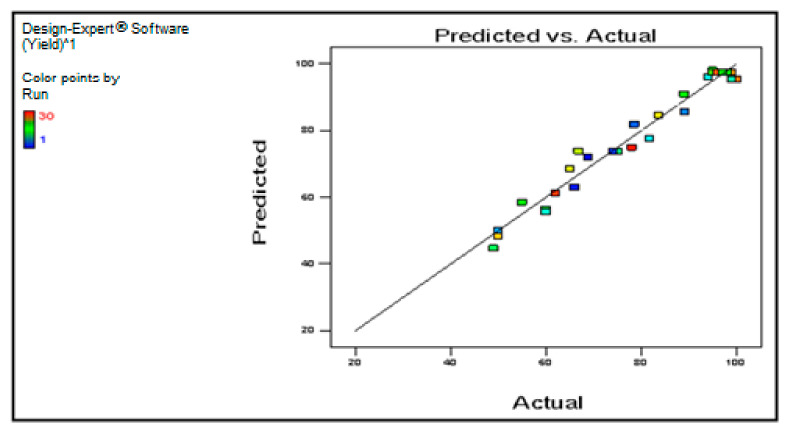
The predicted biodiesel production against the actual output.

Figure 6 depicts the experimental and projected values for the production of biodiesel in a fixed bed membrane reactor, with a good R-square value of 0.9574. The numbers were in close proximity to the 45-degree line, indicating a significant connection between the prediction models and the real data from the study.

**Figure 7:**
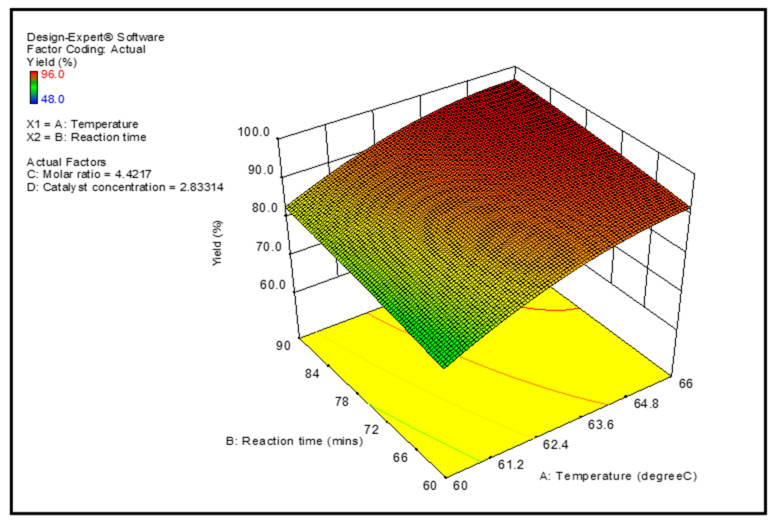
Projected biodiesel production against reaction time and temperature on a response surface 3D graphic.

**Figure 8:**
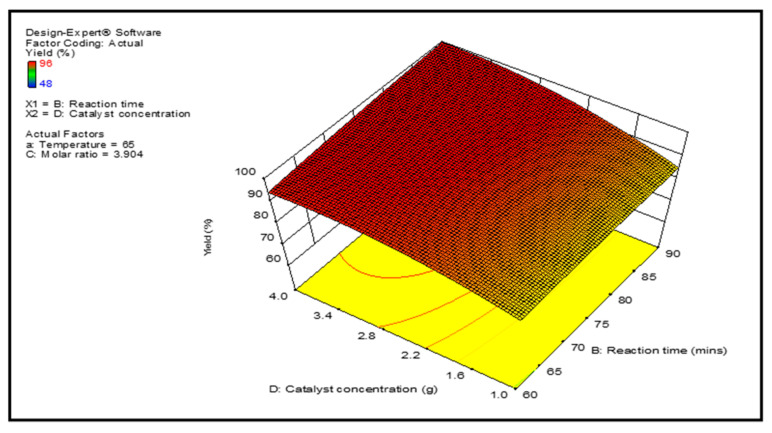
Projected biodiesel production against reaction time and catalysts concentration on a response surface 3D graphic.

**Figure 9:**
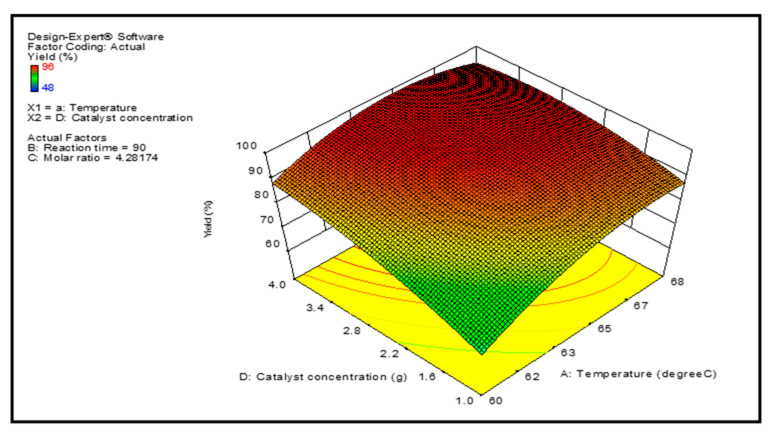
Projected biodiesel production against temperature and catalyst concentration on a response surface 3D graphic.

**Figure 10:**
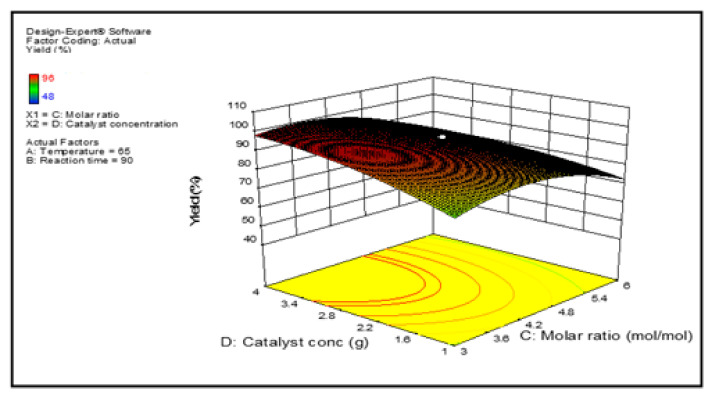
Projected biodiesel production against molar ratio and catalyst amount on a response surface 3D graphic.

Graphical representations of the regression model of reaction parameters are represented by 3-D surface graphs. Figure 7, Figure 8, Figure 9 and Figure 10 show the surface graphs of the biodiesel yields as depicted in the previous section. From A through D, the plots depict the interaction between two independent variables on a single dependent variable, which is the biodiesel yield. The plots are created with the use of the regression model analysis and depict the correlations between each independent variable and the response variables.

Figure 7 depicts the significant interaction between temperature (°C) and reaction time (min), and the change in the biodiesel yield is well shown in the plot; that is, the biodiesel yield increases significantly when both temperature and reaction time are increased. The graph revealed that the rate of conversion of feedstock to product yield grew as the temperature and reaction time increased. This might be owing to the fact that the viscosity of the oil decreases as the temperature rises, resulting in enhanced blending of the feedstock with the methanol and quicker dissociation of the glycerin from the biodiesel mixture. The Arrhenius equation, which predicts that gradual increases in reaction rate constant caused by temperature increase may cause the yield to grow, might be used to explain this phenomenon further. This conclusion is consistent with those described in the literature, according to which a greater reaction temperature and early mixing of the immiscible reactants result in a larger yield of biodiesel being produced [11].

Figure 8 shows the impact of catalyst concentration and reaction time at 65 degrees Celsius and a molar ratio of 4.4:1. The yield of biodiesel rose as the concentration of catalysts and the reaction time increased. However, due to prolong reaction time, there was a little decline in the rate of biodiesel production, which was caused by the impact of the reversible reaction [23]. It can be seen from the three-dimensional response graph that there is a considerable interactions impact between the catalyst concentration and the reaction time on the final Product yield.

The image in Figure 9 shows the feedstock to biodiesel conversion increased when the temperature and catalyst concentration are increased in the process. Conversely, increasing the catalyst amount led to a loss in yields because the catalyst concentration has a detrimental influence on the biodiesel that is generated. The generation of soap during transesterification reaction is responsible for the drop in productivity observed at greater concentrations of the catalyst.

Figure 10 displays the three-dimensional contour map at 90 min and 65 degrees Celsius. With increased methanol: feedstock molar ratio and catalyst amount, the biodiesel production improved moderately. Thereafter, As the molar ratio rises, the yield begins to decline. Usually, a large molar ratio promotes the production of biodiesel fuel and assures the reaction’s completeness. However, because transesterification is a reversible process, an excess of methanol would effectively inhibit the catalyst and revert the process [24].

### 3.3. Optimization Study

The synthesis of biodiesel from soyabean feedstock in the fixed bed membrane reactor was optimized based on the framework that was developed and the input requirements that were used. The primary goal of this research was to employ the use of membrane reactor in the biodiesel synthesis and to optimize the conversion rate of the feedstock to product yield. Table 5 contains a list of all the variables and outputs that have a maximum and minimum range, correspondingly, to fulfill the requirements that have been established for the optimum. To determine the accuracy of the constructed model, a transesterification experiments were conducted under optimal circumstances. The trials were performed by running the coded factors as shown in Table 3. There was less than a one percent product yield difference between the predicted and actual data, showing that the regression designed model performed satisfactorily.

### 3.4. Biodiesel Characterization

The physical and chemical parameters of the synthesized biodiesel were tested in accordance to the ASTM and SANS test methods as follows: viscosity at 40 °C, water content, density at 15 °C, total acid number, total contamination, Sulphur, and flashpoint. Table 6 shows the outcomes of these characterization studies.

The outcome of these tests revealed that the biodiesel generated utilizing membrane technology is in compliance with SANS 1935 and ASTM biofuel criteria. Although there are many biodiesel features that have a direct effect on an engine’s performance, but its viscosity, its high flash point, and its lower density are the most critical. In addition to extending the life of the engine, these features assist to provide better lubrication and full combustion, allowing the output of the engine to increase significantly.

## 4. Summary

In conclusion, RSM experiments were used to find the best conditions for producing biodiesel from soyabean oil feedstock in a membrane reactor in the current study. Due to its high flux and great permeate quality, the TiO_2_/Al_2_O_3_ ceramic membrane reactor with a pore size of 0.02 microns proved to be an excellent choice for the reacting and separating procedures. This process showed that it was possible to synthesize high-quality biodiesel product without the need for further washing and purifying processes which were the limitation found in previous studies. There were substantial impacts discovered for factors such as temperature, reactivity time, molar ratio, and catalyst loading. The maximum conversion, 96.9 percent, was achieved at 65 degrees Celsius, 90 min of reaction time, a 4.2:1 molar ratio, and a catalyst concentration of 3.0 weight percent. The product’s attributes and properties were well within the set criteria of the ASTM and the SANS standards.

## Figures and Tables

**Table 1:** Response Surface Methodology experiment range and parameters.

Parameters	Symbol	−1	0	1
Temperature (°C)	X_1_	60	65	70
Reaction time (minutes)	X_2_	60	90	120
Molar ratio	X_3_	3:1	4:1	6:1
Catalyst concentration	X_4_	1	2.5	4
Output				
Biodiesel yield (%)	Y			

**Table 2:** Characterization of catalyst (CaO/AC) with support.

Analysis	Method	Result
Pore volume	BET	0.152 cm^3^/g
Micro pore volume	BET	0.121 cm^3^/g
Average pore width	BET	2.87 nm
BET surface area	BET	240.51 m^2^/g
Active concentration sites	TPD-CO_2_	1.436 mmol/g

**Table 3:** Experimental matrix results.

Standard Runs	Randomized Runs	Coded Factors				Response Y
		X_1_	X_2_	X_3_	X_4_	
**1**	**29**	−1	−1	−1	−1	62
**2**	**5**	1	−1	−1	−1	90
**3**	**14**	−1	1	−1	−1	60
**4**	**12**	−1	1	−1	−1	75
**5**	**13**	−1	−1	1	−1	49
**6**	**2**	1	−1	1	−1	66
**7**	**18**	−1	1	1	−1	55
**8**	**8**	1	1	1	−1	79
**9**	**24**	−1	−1	−1	1	84
**10**	**27**	1	−1	−1	1	92
**11**	**17**	−1	1	−1	1	89
**12**	**11**	1	1	−1	1	95
**13**	**6**	−1	−1	1	1	50
**14**	**10**	1	−1	1	1	60
**15**	**30**	−1	1	1	1	78
**16**	**3**	1	1	1	1	74
**17**	**25**	−2	0	0	0	50
**18**	**22**	2	0	0	0	60
**19**	**4**	0	−2	0	0	78
**20**	**15**	0	2	0	0	95
**21**	**23**	0	0	−2	0	65
**22**	**21**	0	0	2	0	48
**23**	**1**	0	0	0	−2	62
**24**	**9**	0	0	0	2	94
**25**	**20**	0	0	0	0	93
**26**	**7**	0	0	0	0	94
**27**	**26**	0	0	0	0	92
**28**	**28**	0	0	0	0	95
**29**	**19**	0	0	0	0	93
**30**	**16**	0	0	0	0	96

**Table 4:** ANOVA for Response Surface Quadratic model.

Analysis of Variance Table [Partial Sum of Squares-Type III]						
	Sum of		Mean	F	*p*-Value	
Source	Squares	df	Square	Value	Prob > F	
Model	8183.89	14	584.56	24.05	<0.0001	significant
X_1_: A-Temperature	2412.32	1	2412.32	99.26	<0.0001	
X_2_: B-Reaction time	1199.92	1	1199.92	49.37	<0.0001	
X_3_: C-Molar ratio	310.32	1	310.32	12.77	0.0028	
X_4_: D-Catalyst concentration	933.75	1	933.75	38.42	<0.0001	
X_1_X_2_: AB	31.08	1	31.08	1.28	0.2759	
X_1_X_3_: AC	6.63	1	6.63	0.27	0.6091	
X_1_X_4_: AD	253.61	1	253.61	10.44	0.0056	
X_2_X_3_: BC	303.63	1	303.63	12.49	0.0030	
X_2_X_4_: BD	145.81	1	145.81	6.00	0.0271	
X_3_X_4_: CD	222.76	1	222.76	9.17	0.0085	
X_1_^2^: A^2^	2223.26	1	2223.26	91.48	<0.0001	
X_2_^2^: B^2^	61.97	1	61.97	2.55	0.1311	
X_3_^2^: C^2^	643.77	1	643.77	26.49	0.0001	
X_4_^2^: D^2^	361.05	1	361.05	14.86	0.0016	
Residual	364.54	15	24.30			
Lack of Fit	360.66	10	36.07	46.54	0.1533	Not significant
Pure Error	3.88	5	0.78			
Cor Total	8548.43	29				

**Table 5:** Numerical optimization results and constraints for the factors/response.

Parameter	Goal	Experimental Region		Optimum Condition	
		Lower	Upper	Theoretical Value	Experimental Value
**Temperature (°C)**	In range	60	70	65	65
**Reaction time (min)**	In range	60	120	90	90
**Catalyst concentration**	target	-	3	3	3
**Molar ratio**	In range	3:1	6:1	4.2:1	4.2:1
**Yield (%)**	Maximize			97.7	96.9

**Table 6:** Biodiesel synthesized in a membrane reactor using soybean oil as feedstock characterization [18].

Characteristic	Test	Units	ASTM and SANS 1935 Specification Limit	Result
Density @ 15 °C	ASTM D7042	g/mL	0.86–0.9	0.87
Viscosity @ 40 °C	ASTM D7042	cSt	3.5–5	3.8
Flash point	ASTM D93	°C	120 min	167
Water content	ASTM D6304	%	0.05 max	-
Total acid number	-	mgKOH/g	0.5 max	0.21
Total Contamination	IP 440	mg/Kg	24 max	2
Sulphur	ASTM D4294	ppm	10 max	1

## Data Availability

Data is contained within the article.
